# Comparative cytogenetic study on two species of the genus *Entedon* Dalman, 1820 (Hymenoptera, Eulophidae) using DNA-binding fluorochromes and molecular and immunofluorescent markers

**DOI:** 10.3897/CompCytogen.v6i1.2349

**Published:** 2012-02-23

**Authors:** Nadezhda L. Bolsheva, Vladimir E. Gokhman, Olga V. Muravenko, Alex V. Gumovsky, Alexander V. Zelenin

**Affiliations:** 1 Engelhardt Institute of Molecular Biology, Russian Academy of Sciences, Moscow 119991, Russia; 2 Botanical Garden, Moscow State University, Moscow 119234, Russia; 3 Institute of Zoology, National Academy of Sciences of Ukraine, Kiev 01601, Ukraine

**Keywords:** Hymenoptera, Eulophidae, *Entedon*, chromosomes, karyotypes, base-specific fluorochromes, fluorescence *in situ* hybridization (FISH), DNA methylation

## Abstract

Karyotypes of *Entedon cionobius* Thomson, 1878 and *Entedon cioni* Thomson, 1878 (Hymenoptera: Eulophidae) were studied using DNA-binding ligands with different base specificity (propidium iodide, chromomycin A_3_, methyl green and DAPI; all these ligands, except for the last one, were used for the first time in parasitic wasps), C-banding, fluorescence *in situ* hybridization (FISH) with a 45S rDNA probe and 5-methylcytosine immunodetection. Female karyotypes of both species contain five pairs of relatively large metacentric chromosomes and a pair of smaller acrocentric chromosomes (2n = 12). As in many other Hymenoptera, males of both *Entedon* Dalman, 1820 species have haploid chromosome sets (n = 6). Fluorochrome staining revealed chromosome-specific banding patterns that were similar between the different fluorochromes, except for the CMA_3_- and PI-positive and DAPI-negative band in the pericentromeric regions of the long arms of both acrocentric chromosomes. The obtained banding patterns were virtually identical in both species and allowed for the identification of each individual chromosome. C-banding revealed a pattern similar to DAPI staining, although centromeric and telomeric regions were stained more intensively using the former technique. FISH detected a single rDNA site in the same position on the acrocentric chromosomes as the bright CMA_3_-positive band. Immunodetection of 5-methylcytosine that was performed for the first time in the order Hymenoptera revealed 5-methylcytosine-rich sites in the telomeric, centromeric and certain interstitial regions of most of the chromosomes.

## Introduction

Parasitic wasps are a very diverse, taxonomically complicated and economically important group of insects (Rasnitsyn 1980, Heraty et al. 2011). They attack many insect species, including important agriculture and forestry pests. Parasitic Hymenoptera are therefore widely used in both outdoor and indoor biological plant protection (Quicke 1997, Heraty 2004). The estimated number of potentially described species of parasitic Hymenoptera far exceeds 600,000 (Heraty 2009) or even approaches one million (Quicke 1997), but chromosomal analysis has only been performed on approximately four hundred species (Gokhman 2009). Males of parasitic wasps are usually haploid and females are usually diploid (Crozier 1975). The haploid chromosome number varies from 3 to 23 (Gokhman 2009). Nevertheless, karyotypes of most species of parasitic Hymenoptera have only been studied using routine chromosomal staining. Localization of nucleolus organizing regions (NORs) has been determined using AgNOR staining in only a few species of the superfamily Chalcidoidea (e.g. Reed 1993, Bernardo et al. 2008, Gebiola et al. 2012). However, certain base-specific fluorochromes, such as 4’, 6-diamidino-2-phenylindole (DAPI) and chromomycin A_3_ (CMA_3_), are now widely used to visualize chromosome segments with different AT/GC base compositions in many other insect groups, including aculeate Hymenoptera (Rocha et al. 2002, Kuznetsova et al. 2003, Lachowska 2008, Monti et al. 2010). Surprisingly, parasitic Hymenoptera have never been studied using these techniques. Additionally, fluorescence *in situ* hybridization (FISH) with rDNA probes is also a well-known and highly specific method of revealing nucleolus organizers in many organisms (as demonstrated in Winterfeld and Röser 2007, and Cabrero and Camacho 2008). However, *Trichogramma kaykai* Pinto et Stouthamer, 1997 (Trichogrammatidae) remains the only parasitic wasp species that has been studied using this technique (Van Vugt et al. 2005, 2009).

Currently, it is becoming obvious that DNA methylation plays a crucial role in the regulation of gene activity (Field et al. 2004, Vanyushin 2006, Gehring and Henikoff 2007, Suzuki and Bird 2008). Moreover, the first results of full genomic sequencing of three parasitic Hymenoptera species of the genus *Nasonia* Ashmead, 1904 (Pteromalidae) demonstrated that these parasitic wasps carried genes encoding a full DNA methylation toolkit, including all three types of DNA cytosine-5-methyltransferases (The *Nasonia* Genome Working Group 2010). Nevertheless, DNA methylation patterns of chromosomes have never been studied for these parasitic Hymenoptera, or any other parasitic wasp.

The present study used molecular cytogenetic techniques to examine two closely related species of parasitic Hymenoptera that belong tothe genus *Entedon* Dalman, 1820 of the family Eulophidae. This genus comprises about 170 described species (http://www.nhm.ac.uk/research-curation/research/projects/chalcidoids/database/index.dsml, on February 8, 2012); however, critical revision of the group is needed. Both studied species, namely, *Entedon cionobius* Thomson, 1878 and *Entedon cioni* Thomson, 1878, though being slightly habitually different, belong to the same species group, *Entedon cioni*, and attack larvae of the genus *Cionus* Clairville, 1798 (Coleoptera: Curculionidae) in Europe (Gumovsky 1997; see below). Currently, karyotypes of approximately 60 species of the family Eulophidae have been studied (Gokhman 2002, 2009, Gebiola et al. 2012). Hitherto, only the chromosomes of undetermined species of the genus *Entedon* were examined recently (Gokhman 2004).

## Materials and methods

### Origin of parasitic wasps

Both *Entedon cionobius* and *Entedon cioni* are gregarious endoparasitoids of beetle larvae of the genus *Cionus*. Host larvae potentially containing broods of *Entedon* species were collected on different plants of the genus *Scrophularia* Linnaeus, 1753 (Lamiales: Scrophulariaceae) in Kiev, Ukraine, in May 2010. Weevil larvae were fed with leaves of the host plant in the laboratory until the emergence of the mature parasitoid larvae. For every brood, a few larvae were retained for identification purposes. Larvae that were reared to adults were subsequently identified by A.V. Gumovsky. Voucher specimens were deposited in the collection of the Institute of Zoology of the National Academy of Sciences of Ukraine.

### Preparation of chromosomes

Chromosomal preparations were obtained from cerebral ganglia of prepupae generally following the protocol developed by Imai et al. (1988). Ganglia were extracted from insects dissected in 0.5% (not 1% as proposed by Imai et al. 1988) hypotonic sodium citrate solution containing 0.005% colchicine. The extracted ganglia were then transferred to a fresh portion of hypotonic solution and incubated for 20 min at room temperature. The material was transferred onto a pre-cleaned microscope slide using a Pasteur pipette and then gently flushed with Fixative I (glacial acetic acid: absolute ethanol: distilled water 3:3:4). The tissues were disrupted using dissecting needles in an additional drop of Fixative I. Another drop of Fixative II (glacial acetic acid: absolute ethanol 1:1) was applied to the center of the area, and the more aqueous phase was blotted off the edges of the slide. The same procedure was performed with Fixative III (glacial acetic acid). The slides were then dried for approximately half an hour and stored for about eight months at -20°C.

### Fluorochrome staining

Chromosome spreads were stained with combinations of different fluorochromes, including CMA_3_/DAPI and methyl green (MG)/CMA_3_ (Schweizer and Ambros 1994), propidium iodide (PI)/DAPI (Kim et al. 2002) and MG/DAPI; the latter technique is analogous to distamycin A (DA)/DAPI staining (Donlon and Magenis 1983).

CMA_3_/DAPI staining. The slide was flooded with chromomycin staining solution (0.5 mg/ml in McIlvaine’s buffer (pH 7.0) containing 5 mM MgCl_2_), covered with a coverslip, and incubated at room temperature in the dark overnight. The coverslip was then removed, and the slide was briefly rinsed with distilled water and air-dried. The slide was then flooded with DAPI solution (1 μg/ml in McIlvaine’s buffer), covered with a coverslip, and stained in the dark at room temperature for 15 min. The coverslip was then removed, and the slide was briefly rinsed with distilled water before being air-dried. The preparation was then mounted in a 1:l mixture of glycerol/McIlvaine’s buffer containing 2.5 mM MgCl_2_ and sealed with rubber cement. The slide was aged prior to examination by storing in the dark at 30–37°C for a minimum of one day.

MG/CMA_3_ staining. The slide was stained with CMA_3_ solution (see above) for approximately one hour and then briefly rinsed with distilled water and air-dried. The preparation was then counterstained for l0–20 min with MG solution in McIlvaine’s buffer (3.5 μg/ml), briefly rinsed with distilled water, and then air-dried. The slide was then mounted in glycerol and sealed with rubber cement. The preparation was stored for one day at 37°C prior to analysis by epifluorescence microscopy.

PI/DAPI staining. The slide was stained with a PI and DAPI mixture (1 mg/ml and 0.5 mg/ml, respectively, in McIlvaine’s buffer) for 20 min with 10 min of pre- and post-incubation in McIlvaine’s buffer. The slide was then briefly rinsed with distilled water, air-dried and mounted in VECTASHIELD anti-fading medium (Vector Laboratories).

MG/DAPI staining. The slide was stained with MG solution in McIlvaine’s buffer (0.35 mg/ml) for 15–30 min and rinsed with distilled water and air-dried. The preparation was then stained with DAPI solution (see above) for 5 min in the dark, rinsed with distilled water, and then air-dried. The slide was then mounted in a mixture of glycerol and McIlvaine’s buffer (1:1).

### Fluorescence in situ hybridization (FISH)

Plasmid pTa 71 containing the full DNA sequence of the 45S rRNA gene of wheat was used as the probe for visualizing ribosomal genes (Gerlach and Bedbrook 1979). This probe was labeled using Biotin-Nick Translation Mix (Roche). FISH with rDNA probes was carried out as described previously (Muravenko et al. 2009). Chromosome slides were pretreated with 1 mg/ml RNAse A (Roche) in 2 × SSC at 37°C for 1 h, washed three times for 10 min in 2 × SSC, dehydrated in a series of 70%, 85% and 96% ethanol solutions, and then air-dried. The hybridization mixture contained 50% de-ionized formamide, 10% dextran sulfate, 1% Tween 20 and 2 × SSC. Fifteen microliters of hybridization mixture containing 40 ng of biotin-labeled DNA probe was added to each slide. The slides were then coverslipped, sealed with rubber cement and denatured at 74°C for 5 min. The hybridization was carried out in a moisture chamber at 37°C overnight. After removing the coverslips, the slides were washed twice with 0.1 × SSC at 44°C for 10 min, followed by washing with 2 × SSC at 44°C for 2 × 5 min and with 2 × SSC at room temperature for 5 min. Biotin was detected using avidin-fluorescein isothiocyanate (avidin-FITC) (Vector Laboratories). The slides were mounted in VECTASHIELD anti-fading medium (Vector Laboratories) containing 1.5 μl of DAPI (Sigma-Aldrich).

### C-banding

C-banding was carried out as described by Bolsheva et al. (1984), with some modifications. The slides were incubated in a saturated solution of Ba(OH)_2_ for 6.5 min at room temperature, rinsed in 1 N HCl for 30 sec and then washed with tap water for 15 min. Slides were then incubated in 2 × SSC at 60°C for 1 h, briefly rinsed in distilled water, air-dried, and then stained with 1.5% Giemsa solution (Merck) in 0.075 M phosphate buffer (pH = 6.8) for 1–3 min under control of a light microscope.

### Study of DNA methylation patterns

5-methylcytosine was detected according to the protocol described by Pendina et al. (2005), with a few modifications. Chromosomal preparations were denatured in a 2 M HCl solution at 37°C for 20 min.The preparations were washed twice with ice-cold phosphate-buffered saline (1 × PBS) for 1–2 min and then twice with distilled water, before being air-dried. Then, 100 μl of blocking solution containing 1 × PBS, 5% bovine serum albumin and 0.05% Tween 20 was applied under the coverslips to chromosomal preparations. The preparations were then incubated for 40 min in a moisture chamber at 37°C. After incubation, the slides were quickly rinsed with 1 × PBS containing 0.05% Tween 20. Unconjugated mouse anti-5-methylcytosine monoclonal antibody (American Research Products, Inc.) was dissolved in blocking solution (1:200), and 100 μl was applied to the preparations under the 24 × 60 mm coverslips. The preparations were incubated for 60 min in a moisture chamber at 37°C and then rinsed three times with 1 × PBS containing 0.05% Tween 20 at 43°C for 3 min each. Goat anti-mouse IgG Texas Red-conjugated antibody (Santa Cruz Biotechnology, Inc.) was dissolved in blocking solution (1:200), and 100 μl was applied to the moist preparations under the 24 × 60 mm coverslips. The preparations were then incubated for 40 min in a moisture chamber at 37°C before being rinsed three times with 1 × PBS containing 0.05% Tween 20 at 43°C for 3 min each. The preparations were then quickly rinsed in 1 × PBS, rinsed twice in distilled water, air-dried and then mounted in VECTASHIELD anti-fading medium (Vector Laboratories) containing 1.5 μl of DAPI (Sigma-Aldrich).

### Chromosomal analysis

Metaphase plates were studied and photographed using an Olympus BX-61 epifluorescence microscope fitted with Cool Snap Ropper Scientific black-and-white CCD camera. The obtained images were processed using the VideoTesT-Kario 1.5 software (Ista-VideoTesT, Russia).

## Results

**Figure 1.**
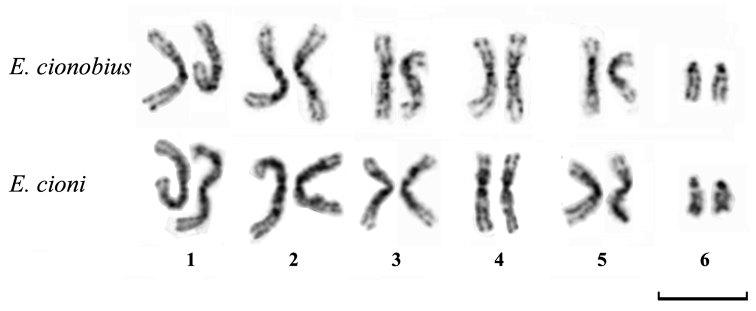
Female karyotypes of *Entedon cionobius* and *Entedon cioni*. Inverted DAPI staining. Bar = 10 μm.

The overall chromosomal morphology of both *Entedon cionobius* and *Entedon cioni* was very similar. Diploid female karyotypes of these parasitic wasps were comprised of five pairs of relatively large metacentric chromosomes (approximately 10 μm in length) and a pair of smaller acrocentric chromosomes (approximately 5 μm) (2n = 12). Males had haploid chromosome sets (n = 6). The DAPI banding patterns in *Entedon cionobius* and *Entedon cioni* were virtually identical. From those patterns, we were able to identify all of the chromosomes within the karyotypes. The DAPI banding patterns demonstrated intensive staining of the pericentromeric regions together with a few weaker interstitial bands on all of the metacentric chromosomes. Additionally, the short arms of the acrocentric chromosomes were also intensively stained (Fig. 1).

**Figures 2–5.**
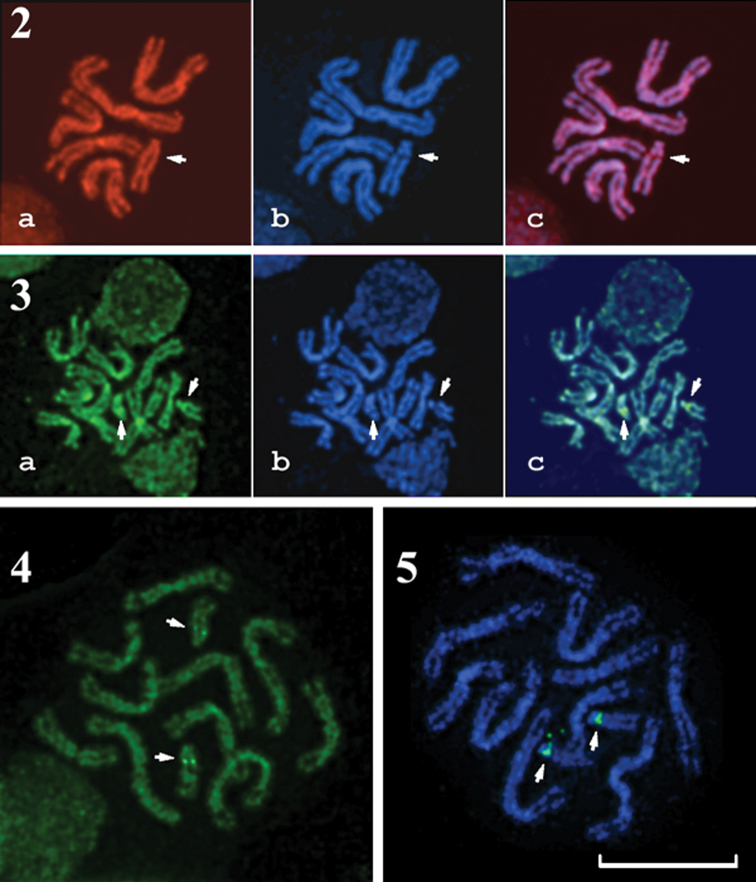
** 2** PI/DAPI-stained male metaphase plate of *Entedon cioni*. **a** PI staining **b** DAPI staining **c** superposition of **a** and **b 3 **CMA_3_/DAPI-stained female metaphase plate of *Entedon cionobius*. **a** CMA_3_ staining **b** DAPI staining **c** superposition of **a** and **b 4** MG/CMA_3_-stained female metaphase plate of *Entedon cionobius*** 5** FISH with 45S rDNA probe on a female metaphase plate of *Entedon cionobius*. Green labels indicate hybridization signals. The chromosomes were counterstained with DAPI. Arrows on Figures 2-5 indicate DAPI-negative, PI- and CMA_3_-positive NORs on acrocentric chromosome 6. Bar = 10 μm.

A similar banding pattern was observed when using PI and CMA_3 _staining. However, there were narrow but distinct pericentromeric DAPI-negative but PI- and CMA_3_-positive bands on the long arms of the acrocentric chromosomes (Figs 2 and 3). CMA_3_/MG staining also demonstrated analogous GC-rich bands in this region (Fig. 4). MG/DAPI staining revealed the same banding patterns as DAPI staining alone (data not shown).

FISH using a 45S rDNA probe (Fig. 5) demonstrated distinct pericentromeric signals on the long arms of the acrocentric chromosomes. Thus, the 45S rDNA is located in the same position as the bright CMA_3_-positive bands that were visualized after CMA_3_/DAPI and CMA_3_/MG staining.

**Figures 6–7.**
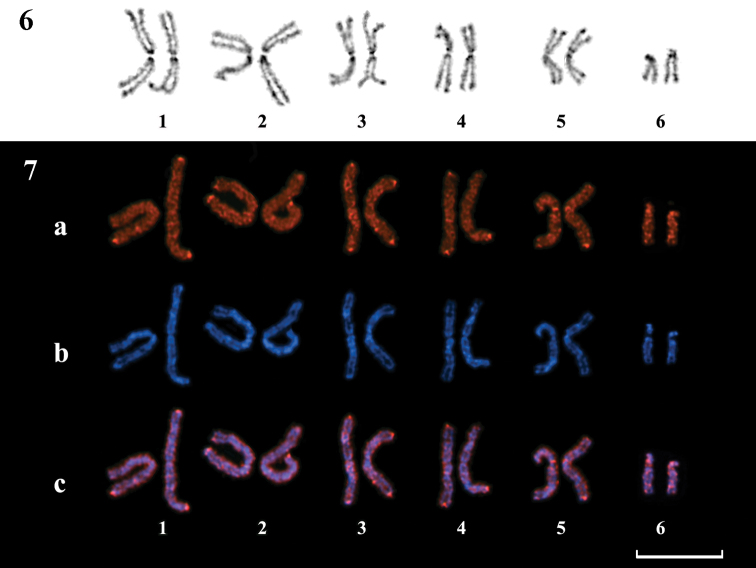
**6** Female karyotype of *Entedon cioni*. C-banding pattern and Giemsa staining** 7** Female karyotype of *Entedon cioni*. Indirect immunodetection of 5-methylcytosine. **a** 5-methylcytosine distribution along chromosomes **b** DAPI counterstaining **c** superposition of **a** and **b**. Bar = 10 μm.

The C-banding pattern (Fig. 6) was analogous to the fluorochrome banding pattern, although it differed in the intensity of staining at the centromeric and telomeric regions. Weakly stained smaller interstitial C-bands were also revealed.

5-methylcytosine immunodetection with fluorochrome-labeled antibodies revealed distinct positive signals in the telomeric regions of most chromosomes. Additionally, a few weaker centromeric and interstitial signals could also be seen. However, the nucleolus organizer region did not demonstrate visible positive signals (Fig. 7).

After DNA denaturation during FISH and 5-methylcytosine detection, DAPI counterstaining demonstrated a banding pattern that was rather similar to both the DAPI- and C-banding patterns (Figs 5, 7). Therefore, it was possible to identify most chromosomes after performing FISH or 5-methylcytosine immunodetection.

## Discussion

The diploid karyotype of the previously studied *Entedon* species (Gokhman 2004) and the karyotypes observed in the species studied in the present paper are rather similar; they comprise five pairs of relatively large metacentric chromosomes and one pair of smaller acrocentric chromosomes. Moreover, this karyotype structure is also characteristic of the overwhelming majority of the family Eulophidae and is therefore considered a ground plan feature of the family Eulophidae (Gokhman 2002, 2004). The banding patterns revealed by DAPI staining on chromosomes of both of the studied species also appeared to be similar, therefore reliably confirming the high karyotypic similarity between the species. All these results are certainly not surprising since both *Entedon cionobius* and *Entedon cioni* belong to the same species group (see above).

Structural heterogeneity of chromosomes is characteristic of many animals, including insects (Rodionov 1999). Staining of mammalian chromosomes with DAPI, an AT-specific DNA-binding ligand, reveals banding patterns that are similar to the regular Q- and G-banding patterns. However, staining with GC-binding ligands revealed R-banding patterns that were inverse to the G-banding ones (Sumner 1994). G-like banding patterns were observed after the regular G-banding procedure of trypsin pretreatment and Giemsa staining in parasitic wasps that belong tothe genus *Encarsia* Förster, 1878 (Aphelinidae) (Odierna et al. 1993, Baldanza et al. 1999). However, the nature of those banding patterns remained obscure. To study the characteristics of DAPI banding obtained during the present investigation, we performed chromosomal staining, both singly and in combination, with DNA-binding ligands of different specificity. We found that DAPI, an AT-binding ligand, CMA_3_, a GC-binding ligand, and propidium iodide, a fluorochrome without preferential affinity for AT or GC pairs, produced the same banding patterns in all chromosomal regions, except for the NOR. Consecutive staining of human or other mammalian chromosomes with DAPI and AT-specific non-fluorescent chemicals, such as distamycin A or methyl green, was reported to produce specific banding patterns that are different from the regular DAPI patterns (Donlon and Magenis 1983, Schweizer and Ambros 1994).Although the molecular mechanisms of this staining are unclear, DA is generally considered to be more effective at displacing DAPI when the latter is bound to contiguous AT clusters instead of mixed AT/GC sequences (Burckhardt et al. 1993). Nevertheless, we found no difference between MG/DAPI and DAPI staining in the species studied. Thus, unlike the characteristic banding patterns of mammalian chromosomes, different fluorochromes produced similar banding patterns on the chromosomes of the two studied *Entedon* species. The patterns observed in the present study indicate differences in packing density between the various chromosomal regions rather than changes in AT/GC composition.

The rDNA sites are highly conserved in all eukaryotic organisms (Muravenko et al. 2001, Smit et al. 2007). Van Vugt et al. (2005, 2009) used a 45S rDNA probe originating from a wheat genome (Gerlach and Bedbrook 1979) to successfully visualize nucleolus organizers in the parasitic wasp *Trichogramma kaykai*. In the present work, FISH using the same 45S rDNA probe revealed the only DAPI-negative and PI- and CMA_3_-positive region in the studied species’ chromosomes, and this region appears to be a NOR. This NOR is localized on the long arm of the sixth acrocentric chromosome, close to the centromere. The position of the NOR in the pericentromeric region of subtelocentric or acrocentric chromosome is typical for parasitic Hymenoptera of the superfamily Chalcidoidea. Nucleolus organizers have been localized on acrocentric chromosomes in a few species of the genus *Encarsia* (Aphelinidae) (Giorgini and Baldanza 2004), in the *Pnigalio soemius* (Walker, 1839) species complex and in *Pnigalio vidanoi* Navone, 1999 of the family Eulophidae (Bernardo et al. 2008, Gebiola et al. 2012). Moreover, localization of NORs on subtelocentric or acrocentric chromosomes was also detected in *Cotesia congregata* (Say, 1836) of the family Braconidae (Ichneumonoidea) (Belle et al. 2002). In all these cases, the nucleolus organizers were localized on the shorter arms of subtelocentric/acrocentric chromosomes. However, in *Melittobia australica* Girault, 1912 (Eulophidae), the nucleolus organizer was localized on the telomere of a large metacentric chromosome (Maffei et al. 2001). The karyotypes of the above-mentioned species bear a single NOR, whereas *Trichogramma kaykai* has two NORs with terminal localization on metacentric chromosomes 1 and 4 (Van Vugt et al. 2005, 2009). These results suggest that further studies are needed to adequately describe the real diversity in the number and localization of nucleolus organizers in parasitic Hymenoptera species.

Currently, 5-methylcytosine localization has been attempted only for mammalian and plant chromosomes (Miller et al. 1974, Schnedl et al. 1975, 1976, Bernardino et al. 2000, Ruffini Casteglione et al. 2002, Cremonini et al. 2003). However, DNA methylation is widespread in social Hymenoptera (Kronforst et al. 2008) where it can mediate nutritional control of caste determination (Kucharski et al. 2008). Nevertheless, the only successful attempt to investigate DNA methylation in insect chromosomes using specific antibodies was reported for the polytene chromosomes of certain Diptera, namely, *Drosophila* Fallen, 1823 and *Sciara* Meigen, 1803 (Eastman et al. 1980).

In many organisms where the distribution of 5-methylcytosine on chromosomes has been studied, high levels of DNA methylation in heterochromatic segments were detected, usually in telomeric and centromeric regions. The occurrence of intense DNA methylation in the analogous heterochromatic regions of *Entedon* chromosomes is therefore not surprising. However, highly functionally active chromosomal regions, such as NORs, are not intensively methylated in many organisms (Santoro and Grummt 2001), including the species here studied.

## Conclusion

The present study supports previous hypotheses and provides new insights into the chromosomal structure of parasitic Hymenoptera. Specifically, several different DNA-binding ligands, such as propidium iodide, chromomycin A_3_ and methyl green, were used for the first time for cytogenetic study of these insects. However, the similarity in banding patterns obtained through these ligands confirms that, unlike in mammals and some other groups, the bands represent differences in packing density along chromosomes instead of differences in base composition. The karyotype structure of the *Entedon* species, and the family Eulophidae in general, appeared to be relatively conserved. However, we were able to demonstrate an unusual position for the nucleolus organizer in both examined species. For the first time in the order Hymenoptera, our data also visualize the presence of 5-methylcytosine in *Entedon *genomes in detectable amounts and its non-random distribution along the chromosomes. This allows the use of 5-methylcytosine immunodetection for the investigation of sex determination, cell differentiation and epigenetic regulation of Hymenoptera genomes.
